# Simplification of vector communities during suburban succession

**DOI:** 10.1371/journal.pone.0215485

**Published:** 2019-05-01

**Authors:** Meredith R. Spence Beaulieu, Kristen Hopperstad, Robert R. Dunn, Michael H. Reiskind

**Affiliations:** 1 Department of Entomology and Plant Pathology, North Carolina State University, Raleigh, North Carolina, United States of America; 2 Department of Applied Ecology, North Carolina State University, Raleigh, North Carolina, United States of America; University of Maryland, UNITED STATES

## Abstract

Suburbanization is happening rapidly on a global scale, resulting in changes to the species assemblages present in previously undeveloped areas of land. Community-level changes after anthropogenic land-use change have been studied in a variety of organisms, but the effects on arthropods of medical and veterinary importance remain poorly characterized. Shifts in diversity, abundance, and community composition of such arthropods, like mosquitoes, can significantly impact vector-borne disease dynamics due to varying vectorial capacity between different species. In light of these potential implications for vector-borne diseases, we investigated changes in mosquito species assemblage after suburbanization by sampling mosquitoes in neighborhoods of different ages in Wake County, North Carolina, US. We found that independent of housing density and socioeconomic status, mosquito diversity measures decreased as suburban neighborhoods aged. In the oldest neighborhoods, the mosquito assemblage reached a distinct suburban climax community dominated by the invasive, peridomestic container-breeding *Aedes albopictus*, the Asian tiger mosquito. *Aedes albopictus* is a competent vector of many pathogens of human concern, and its dominance in suburban areas places it in close proximity with humans, allowing for heightened potential of host-vector interactions. While further research is necessary to explicitly characterize the effects of mosquito community simplification on vector-borne disease transmission in highly suburbanized areas, the current study demonstrates that suburbanization is disrupting mosquito communities so severely that they do not recover their diversity even 100 years after the initial disturbance. Our understanding of the community-level effects of anthropogenic land-use change on arthropod vectors will become increasingly important as we look to mitigate disease spread in a global landscape that is continually developed and altered by humans.

## Introduction

The world is becoming increasingly urban. As recently as 1950, urban areas were occupied by just 30% of the world’s population. Today, 55% of people live in urban areas, with the proportion of people living in cities projected to be as high as 68% by 2050 [1]. In some regions, nearly all urban dwellers live in relatively high-density cities. But in others, a large proportion of human homes are at the margins of cities, in suburbs. Suburban development is the fastest growing anthropogenic land-use in the United States, expanding approximately seven- to ten-fold from 1950–2000 [2]. In line with national trends, this phenomenon is rapidly changing the southeastern United States, and models predict a continued growth in urban and suburban land-use of 101% to 192% over the next 50 years [3]. This land-use change due to anthropogenic development simultaneously disfavors species dependent on wild forests and grasslands, and favors species associated with urban areas, with suburban areas having the potential to act as an ecotone between urban and natural areas or to be completely distinct from natural areas. Among the species suburban and urban development may favor are arthropod species able to vector human pathogens [4].

Vector-borne diseases are serious public health threats, affecting more than one billion people yearly [5] and causing significant health impacts and economic burdens. Globally, mosquitoes are the arthropod disease vectors of greatest importance, transmitting pathogens that cause significant morbidity and mortality such as malaria, dengue virus, the recently emerging Zika virus, and filarial parasites [6]. In recent years, the geographic ranges of many vector-borne diseases have been expanding, increasing their already significant public health and economic impacts. Many factors are potentially contributing to the expansion of vector-borne disease ranges, such as human travel, transport of products, and global climate change, but also anthropogenic land-use changes, including urbanization [7]. Land use changes are of particular interest because of their ability to affect entire species assemblages. Changes in species assemblage can significantly affect the diversity and composition of mosquito species and their relative abundance, all of which have important implications for the spread of vector-borne diseases [8–10].

When natural landscapes are converted to suburban development, the accompanying land clearing and construction acts as a large disturbance event in the environment, impacting species assemblages. Some ecological studies suggests that intermediate levels of disturbance will result in increased diversity, both because disturbance introduces habitat heterogeneity and because disturbance can reduce the abundance of dominant species, increasing potential for invasion [11]. Within the urban setting, this equates to a theoretical diversity peak at intermediate levels of development, when the biotic limitations of rural areas and the physical limitations of urban areas are both alleviated [12]. Bird diversity in Twin Cities, Minnesota and Chicago, Illinois, for instance, is highest in moderately disturbed areas and then subsequently declines in highly urban areas [13,14]. Similarly, butterfly diversity in an area of former oak woodlands in Palo Alto, California is lowest in the most urbanized areas, but in this case, even moderate levels of land development are detrimental to the natural species assemblage despite the overall intermediate disturbance diversity peak [12]. It is currently unclear whether the fine-scale heterogeneity in land-use associated with suburban development may lead to increases in mosquito biodiversity compared to that in natural woodlands or grasslands, as is seen with other species [15–17].

While understudied, the issue of mosquito species diversity in disturbed areas is important when considering the spread of vector-borne diseases. Vector competence for a given pathogen varies between species, leading to obvious implications for disease risk after community-level changes. Changes in the diversity or evenness of a community could affect vector-borne disease transmission if the species assemblage is shifted toward one dominated by mosquitoes with greater vectorial capacity. This is particularly relevant in suburban areas, as these disturbed environments place vector mosquitoes in close proximity and routine contact with hosts, including humans and their companion animals, which may increase risk for disease spread [4,18].

Despite the rise in urbanization and its potential effects on mosquito communities, and a rapid increase in the number of studies of the effects of urbanization, few studies have considered the ways in which urbanization in general, and suburbanization in particular, influence the overall composition of mosquito communities (S1 Fig). Where the influence of habitat on mosquito communities has been studied, it is most often in the context of exclusively natural habitats. For example, studies investigating the species assemblages present at wooded sites when compared to pasture or grassland sites have shown, perhaps unsurprisingly, that these habitats tend to sustain different mosquito species [8,19,20]. These links between mosquito species and habitat can arise because of adaptation to certain habitats through specificity in breeding sites or selectivity of use by adult mosquitoes, with fine scale (< 20m) variation in flying, host-seeking, and adult distribution [21]. In contrast to the amount of studies on mosquito assemblages in natural areas, those investigating similar questions in urbanized areas are less common. The studies that have considered primarily anthropogenic landscapes have tended to focus on species-specific distributions rather than communities as a whole (e.g. [22–24]). The few studies that have approached questions of community-level effects have had study sites that are vastly different from suburban areas in the United States (e.g. [9,25]), leaving a gap in knowledge as to the effects of suburbanization on mosquito assemblages. Given that suburban areas are heterogeneous landscapes composed of grass, shrubs, trees, man-made structures, and perhaps surrounded by undeveloped natural areas, it is unclear whether mosquito assemblages present in these areas are more like those in fields, woodlots, or something unique to the suburban landscape.

Here we sought to characterize the changes to mosquito assemblages that occur after human driven land-use change in the context of suburban development, and, if such changes exist, the rate at which these changes occur post-development. We hypothesize that 1) suburban species assemblages are distinct from those in either undeveloped fields or undeveloped woodlots, and 2) the mosquito assemblage changes rapidly after the initial disturbance, approaching a suburban climax community through time. We tested these *a priori* predictions by establishing a chronosequence of suburban developments in which to sample mosquitoes, and comparing these species assemblages to those present in uncultivated field and woodlot areas.

## Methods

### Study overview

We conducted this research in Wake County, North Carolina, USA. Wake County has a temperate climate and consists of a major urban center, Raleigh, and extensive suburbs, making it North Carolina’s second most populous county with around 1 million residents [26]. To characterize the changes in mosquito assemblage that occur after human driven land-use change, we sampled mosquitoes in neighborhoods that were previously woodlots before development as well as neighborhoods that were fields prior to development, based upon historical aerial photos in Google Earth [27]. To compare the community assemblages to those in natural areas, we also sampled mosquitoes in natural woodlots and natural fields and grasslands as controls. To define the rate at which any changes to the mosquito assemblage occur after suburban development, we established a chronosequence by sampling in neighborhoods of various ages. This approach allowed us to determine the effects of approximate time scales within a two-year study.

### Site selection and owner permission

**Fig 1:**
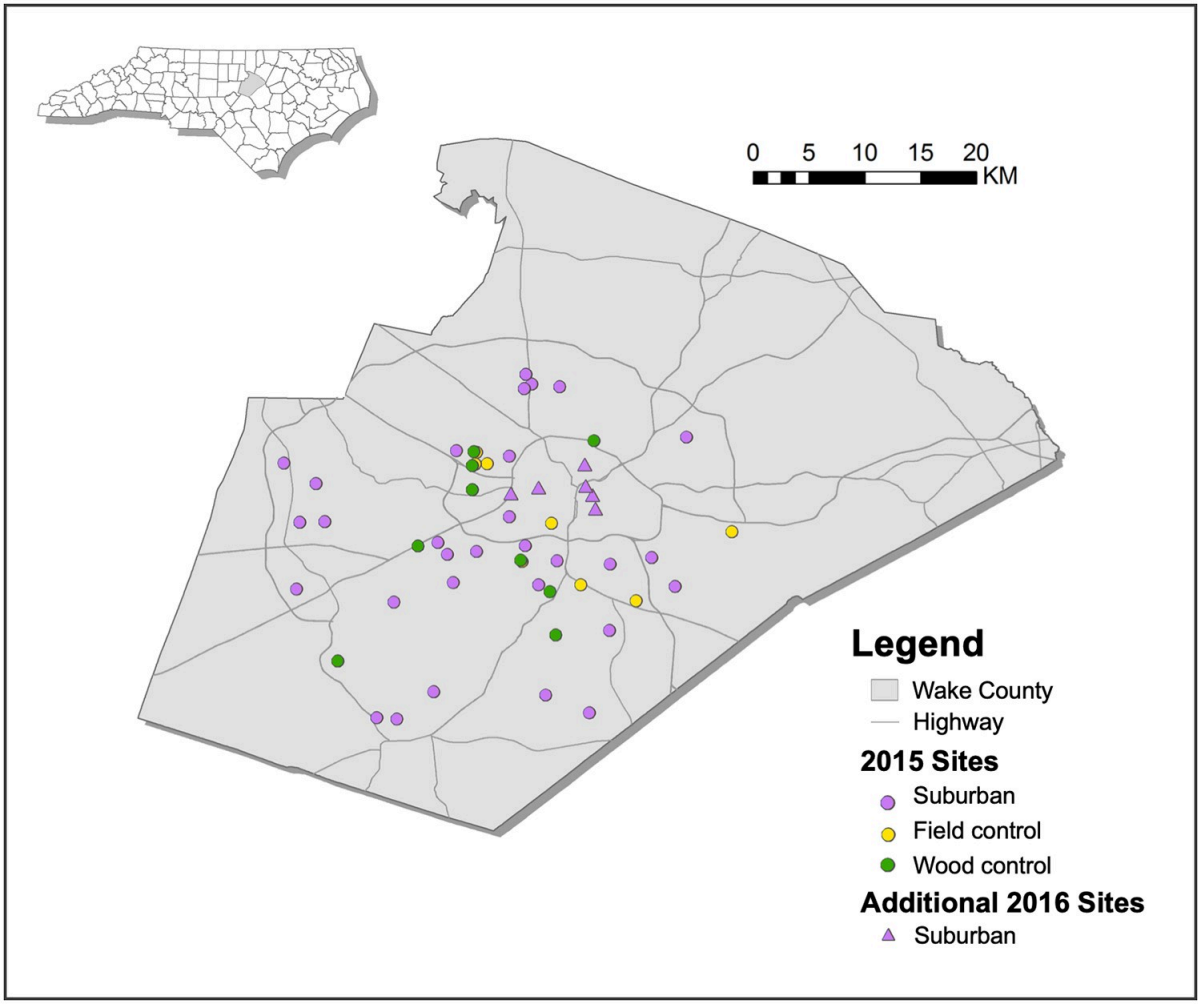
Trapping sites within Wake County, North Carolina. All 2015 sites denoted by a circle were also sampled in 2016, with the addition of 6 older suburban sites that were sampled exclusively in 2016, denoted by a triangle. Map created using public domain data from Wake County Government’s Wake County GIS [28] and the U.S. Census Bureau’s 2017 TIGER/Line Shapefiles [29].

We identified candidate suburban neighborhoods using Google Earth current and historical imagery. We defined suburban neighborhoods as those consisting of detached single-family homes not located on a city block. Based on historical images, we categorized candidate neighborhoods in Wake County, NC by age and then classified them as previously fields or previously woodlots before development. To ensure that neighborhoods of various ages were being represented in the study, we created age categories to guide neighborhood selection: developed before 1993, between 1993 and 2002, between 2003 and 2007, between 2008 and 2012, and from 2013 to present. As changes in mosquito assemblage were predicted to happen fairly quickly after the initial disturbance, age category intervals were designed to be shorter when neighborhood development was closer to the present time, and longer when development was further removed. This allowed us to better capture periods of predicted rapid change in our chronosequence, while reducing sampling efforts in periods of less rapid change. Within the age categories, we selected neighborhoods from each previous land use (i.e. fields or woodlot). To ensure even sampling throughout Wake County, we split the county into geographical quadrants and selected neighborhoods across all quadrants in a haphazard manner. Because of the stringent requirements of neighborhood age, previous land use, and the necessity for homeowner permission for trapping, site selection was fairly limited, but each geographical quadrant had at least one site representative of each age category and previous land use. Overall, we selected 30 neighborhoods in 2015 that spanned the age categories and previous uses from across Wake County (Fig 1). Within each neighborhood, we selected a single house for trap placement based on homeowner approval. We verified that the homeowner did not intend to perform any mosquito pesticide applications during the course of the study. Location of the trap within the yard was based entirely on homeowner preference, although the majority of homeowners elected to have the trap placed in their backyard near the property edge.

We also sampled undeveloped field and woodlot habitats as controls. We used three natural habitat sites: Schenck Memorial Forest at North Carolina State University (NC State), NC State’s Equine Educational Unit, and NC State’s Lake Wheeler Beef Unit. Each of these natural sites featured both field habitat and woodland habitat, and we placed traps at least 100m away from a habitat edge, consistent with previous findings of mosquito habitat fidelity [21]. As large undisturbed wild areas are not especially common around Raleigh, NC, we also sampled at smaller parcels of land consisting of natural woodlots and natural fields as control sites. Each of these smaller control sites had at least a 100m radius of non-developed natural land around the trap, again consistent with previous findings of mosquito habitat fidelity [21]. We sampled at six of these smaller wooded control sites and five smaller field control sites, giving us an overall total of 9 woodlot control sites and 8 field control sites.

We repeated the study in 2016, trapping at all of the same control sites and at different houses within the same neighborhoods that were sampled in 2015. Given that the oldest neighborhood that we sampled in 2015 was 40 years old, we also added six additional older neighborhoods that were developed between 50 and 102 years prior in 2016, resulting in a total of 36 neighborhoods sampled (Fig 1) and giving us an even broader time scale for chronosequence analysis.

### Trapping

We used CDC light traps (JW Hock Co., Gainesville, FL) to sample mosquitoes throughout the course of the study. We removed the lights from the CDC light traps to reduce by-catch, and baited the traps with a small cooler containing 1kg of dry ice (solid CO_2_) to attract host-seeking mosquitoes. We set traps at each of the neighborhood and control sites for approximately 16 hours overnight biweekly from June through mid-October in 2015 and June through the end of October 2016.

### Specimen identification

We enumerated and identified all collected mosquitoes to species using Burkett-Cadena’s *Mosquitoes of the Southeastern United States* [30] and Darsie and Ward’s *Mosquitoes of North America* [31] dichotomous keys. We then preserved the mosquitoes in 95% ethanol for future reference.

### Land-use classification

We characterized site habitats by their current land use and vegetative structure within 100m radius of the trap. In the geographic information system (GIS) software ArcMap [32], we hand digitized the landscape using contemporary imagery and classified the resultant polygons as one of 9 categories: deciduous forests and evergreen forests (coarse vegetation), scrub/shrub (medium vegetation), grassland (fine vegetation), barren land (bare soil), buildings and pavement (impervious surfaces), cultivated crops, and water. We considered cultivated crops and grassland as field components and classified deciduous and evergreen forests as woodlot components. In the data analysis, we used field components, woodlot components, scrub/shrub, buildings, and pavement as predictor variables and the remaining land-use categories as covariables that were not of immediate interest.

### Statistical analyses

As we sampled at the same neighborhoods and control sites in both years with the addition of six unique older neighborhoods in 2016, we chose to treat the two years separately rather than combining into a single dataset. This allowed us to avoid pseudoreplication of the overlapping neighborhoods between years, verify findings from the first year of trapping, and assess for the effects of adding in neighborhoods greater than 40 years old in the second year of trapping.

All statistical analyses were completed using the Vegan package in R 3.2.2 statistical software [33]. Species accumulation curves were performed to validate that there were enough trapping sites to ensure sufficient mosquito sampling. Recognizing that each technique for measuring diversity has limitations, we approached our analysis of mosquito diversity at each site holistically by measuring rarefied species richness, Pielou’s evenness index, and Shannon-Wiener diversity index. We chose the Shannon-Wiener index due to its widespread use in comparable published studies, as well as its recognition as a valid expression of species diversity [34]. We calculated rarefied species richness, Pielou’s evenness, and Shannon-Wiener diversity index and compared these diversity measures between control woodlots, control field sites, and neighborhood age categories via ANOVA to test the hypothesis that natural sites differ from suburban sites with regard to their mosquito diversity. We performed linear regressions modeling the effects of neighborhood age, housing density, home price, and median household income on rarefied species richness, evenness, and Shannon-Wiener diversity index. Although we are primarily interested in the effects of neighborhood age, housing density and socioeconomic factors could also affect mosquito diversity and covary with neighborhood age. For housing density measurements, we simply counted the number of houses within 100m radius of the trapping site. To approach socioeconomic status, we acquired median household income data by census tract from the 2013 American Community Survey via the United States Census Bureau’s Census Explorer tool [35]. Recognizing that census tracts are much larger than a given neighborhood, we also approached socioeconomic status at the neighborhood scale by calculating the average estimated home price of the trapping site and its two nearest neighbors using Zillow [36], then normalized all values to the average home price in Wake County [37]. We calculated variance inflation factors to assess for collinearity between neighborhood age, housing density, and both measures of socioeconomic status.

We also performed Partial Canonical Correspondence Analysis (PCCA) in R to investigate the relationship between land-use classification and mosquito assemblages for a given site. PCCA is a method for exploring relationships between two multivariate sets of variables measured on the same individual [38]. Here, the individual on which we are measuring our variables is trapping site, and the two multivariate sets of variables are the mosquito species assemblages and the GIS habitat classifications of the land use and vegetative structure around the trapping site. This approach allows us to summarize the relationships between these factors using a lesser number of statistics while preserving the main facets of the relationship. Utilizing the GIS land use classifications described above, we used field components, wood components, scrub/shrub, buildings, and pavement as predictor variables in the PCCA analysis, given our *a priori* assumptions of their effects on mosquito habitats. We controlled for the barren land and permanent water components by treating them as covariates, as their effects were presumed to be minimal when compared to the other land use classification categories. We plotted the CCA axis values against neighborhood age for each of the suburban sites to visualize the effect of neighborhood age in conjunction with the site’s land-use structure and mosquito species assemblage. To complement the PCCA, we also performed analysis of similarities (ANOSIM) using Bray-Curtis dissimilarity to compare the community composition between suburban, field control, and wood control sites. Finally, we utilized similarity percentages analysis (SIMPER) to evaluate the contribution of individual species to the overall Bray-Curtis dissimilarity.

## Results

The species accumulation curves appeared to reach an asymptote for both years, indicating that our trapping sites were adequate to sufficiently sample mosquitoes in this area (S2 Fig). In total, we trapped 22 species of mosquitoes (out of 28 known to be historically present in Wake County, NC [B. Byrd, personal communication, February 8, 2019]) and a total of more than 10,000 individuals. In 2015, we trapped a total of 20 species and 4269 individual mosquitoes, 47.1% of which were the Asian tiger mosquito, *Aedes albopictus*, an invasive species in the U.S. Similar numbers were seen in 2016, with 19 total species and 5975 total individuals, 38.1% of which were our most commonly trapped species, *Ae*. *albopictus*.

Two trapping sites in 2015, one suburban site and one field control site, were subjectively different from the other sites. The suburban site’s trap had been placed close to the home’s HVAC unit at the owner’s request, deviating from the typical back-of-yard placement at other suburban sites. The field site was agricultural land as opposed to the other field controls, which were pasture or unused land. In 2016, one field control site suffered from loss of the land owner’s trapping permission after a single trap night. These three sites yielded fewer than 10 total individuals across the entire summer’s trapping efforts. All of the aforementioned factors could have resulted in lower mosquito yields and made the sites sufficiently different for exclusion, so the three sites were excluded from further analysis.

**Fig 2:**
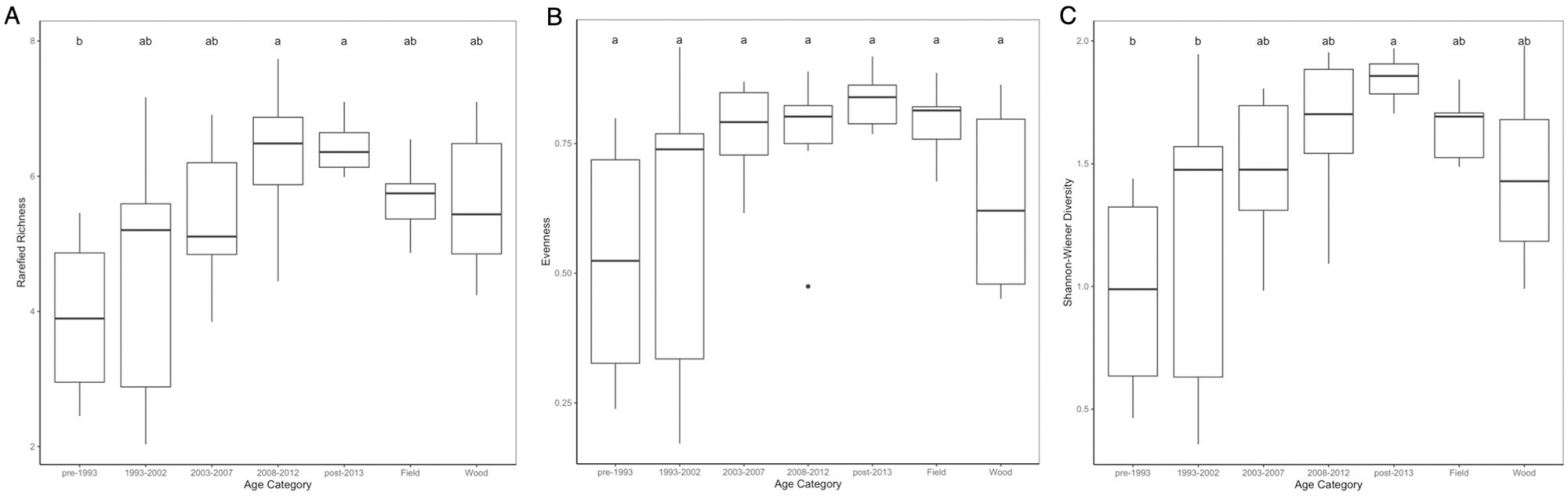
Comparison of diversity measures between control sites and suburban sites by age category in 2015. For all boxplots, the median is represented by the thick black line. Lower and upper hinges denote the first and third quartiles respectively, while whiskers extend from the hinge to the furthest value that is within 1.5x the interquartile range. Outliers are plotted individually. Boxplots are presented for (A) rarefied richness (p = 0.013, df = 6, F = 3.156), (B) evenness (p = 0.032, df = 6, F = 2.614), and (C) Shannon-Wiener diversity (p = 0.007, df = 6, F = 3.561), with significant comparisons denoted by letter above the boxplots.

**Fig 3:**
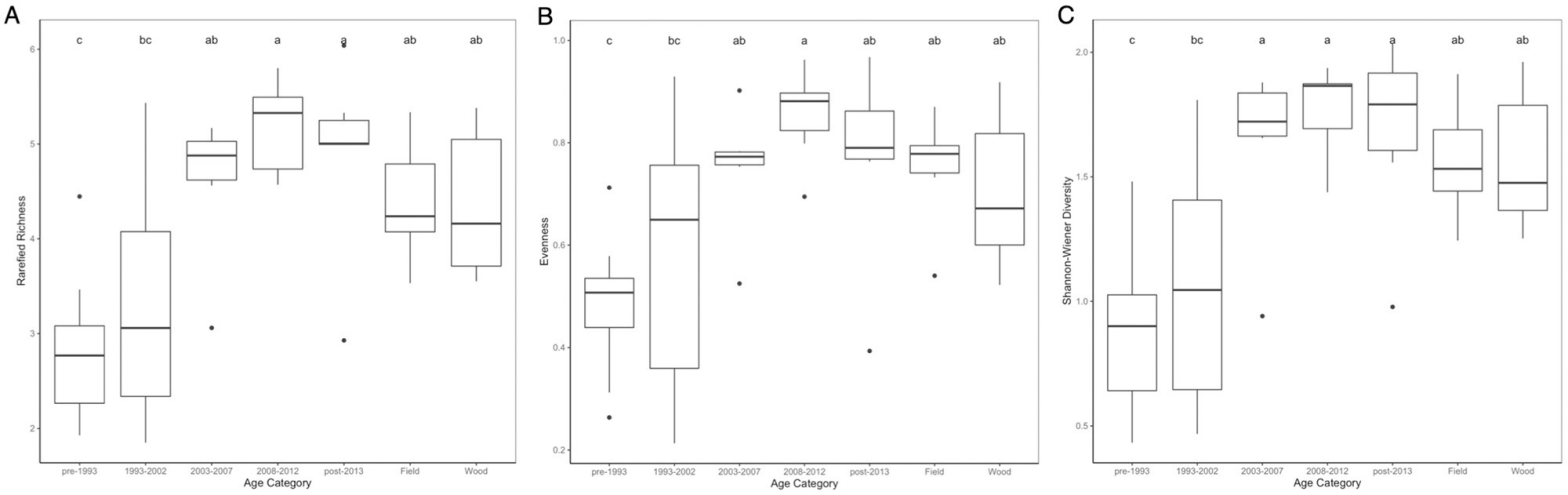
Comparison of diversity measures between control sites and suburban sites by age category in 2016. For all boxplots, the median is represented by the thick black line. Lower and upper hinges denote the first and third quartiles respectively, while whiskers extend from the hinge to the furthest value that is within 1.5x the interquartile range. Outliers are plotted individually. Boxplots are presented for (A) rarefied richness (p < 0.001, df = 6, F = 5.25), (B) evenness (p < 0.001, df = 6, F = 5.25), and (C) Shannon-Wiener diversity (p < 0.001, df = 6, F = 8.655), with significant comparisons denoted by letter above the boxplots.

Rarefied species richness, evenness, and Shannon-Wiener diversity all differed significantly when comparing the natural sites to the suburban sites grouped according to their age categories in 2015 and in 2016. In 2015, the suburban neighborhood age categories differed only among themselves with regard to all diversity measures, and were not significantly different from either of the natural categories (Fig 2). The overall model for rarefied richness was significant (p = 0.013, df = 6, F = 3.156), with the oldest suburban neighborhoods being significantly different from the two youngest age categories. No significant differences found between suburban neighborhoods and control sites. Despite the overall model being significant (p = 0.032, df = 6, F = 2.614), none of the categories were significantly different in their evenness after multiple comparison correction. The two oldest suburban age categories significantly differed from the youngest suburban category with regard to Shannon-Wiener diversity (overall model p = 0.007, df = 6, F = 3.561). Again, no significant differences were found between suburban neighborhoods and control sites. However, in 2016, the oldest neighborhood age category (pre-1993 development) significantly differed from both the natural field and the natural woodlots with regard to all three diversity measures (Fig 3). Various suburban age categories differed when compared with other suburban age categories with their rarefied richness, but of interest is the significant difference between the oldest suburban age category and both the field and wooded control sites (overall model p < 0.001, df = 6, F = 8.173). As with rarefied richness, evenness significantly differed between the oldest suburban sites and both of the control categories, as well as between various of the suburban age categories (overall model p < 0.001, df = 6, F = 5.25). Similar trends were seen with Shannon-Wiener diversity, with the oldest suburban sites significantly differing from both of the control categories being the comparison of interest (overall model p < 0.001, df = 6, F = 8.655).

**Fig 4:**
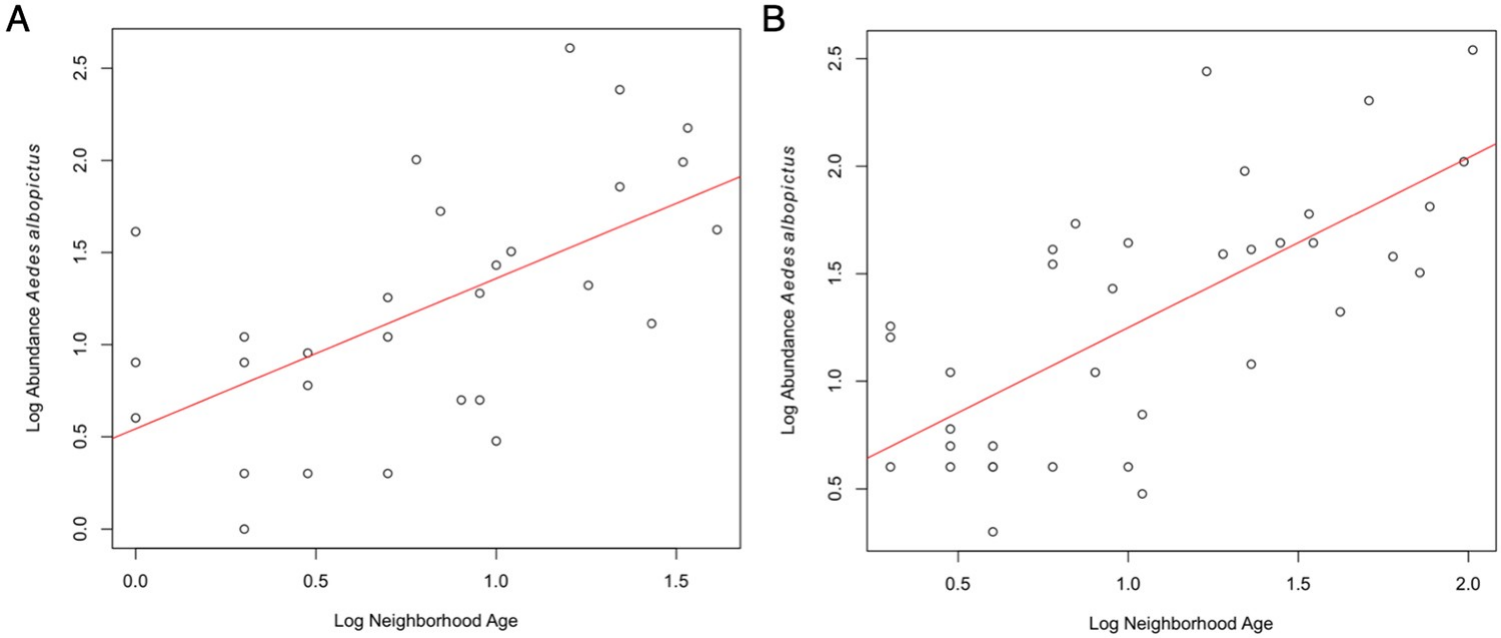
Log abundance of *Aedes albopictus* increases with log neighborhood age. Control field and woodlot sites were excluded from this analysis to focus on the effect of suburban development as these areas become further established. (A) In 2015, a significant positive correlation was noted (p < 0.001, df = 1 and 27, F = 14.3, R^2^ = 0.346, ρ = 0.614). (B) With the addition of older neighborhoods in 2016, a similar significant positive correlation was noted (p < 0.001, df = 1 and 34, F = 31.86, R^2^ = 0.484, ρ = 0.67).

When considering the suburban sites specifically, rarefied richness, evenness, and Shannon-Wiener diversity index were all well explained by a model including neighborhood age, housing density, home price, and median household income for both years of sampling (p ≤ 0.011 for all years and diversity measures, Table 1). When considering these factors individually, neighborhood age was significant in all models (p ≤ 0.005 for all models) and was the only significant factor across both years for any of the diversity measures (Table 1). As neighborhoods age, they become less species rich, less diverse, and species are less evenly distributed. Since *Ae*. *albopictus* made up a significant portion of the collected mosquitoes in both years, we performed a linear regression of *Ae*. *albopictus* abundance versus the primary determinant of diversity changes, neighborhood age. *Aedes albopictus* abundance increased as neighborhoods got older, with neighborhood age explaining 34.6% of variation in *Ae*. *albopictus* abundance in 2015 and 48.4% of variation in 2016 (p < 0.001, Fig 4). The remaining four most abundant species were *Ae*. *vexans*, *Culex pipiens*, *Cx*. *salinarius*, and *Cx*. *erraticus* in 2015, and *Cx*. *salinarius*, *Ae*. *vexans*, *Ae*. *atlanticus*, and *Cx*. *erraticus* in 2016 (S1 Table). Of these species, the only significant relationship consistent across both years was a negative correlation between *Cx*. *erraticus* abundance and neighborhood age (p ≤ 0.001, S3 Fig).

**Table 1:** Effects of neighborhood age, housing density, home price, and household income on mosquito diversity measures.

Year	Diversity Measure	Model p-value, df, F-statistic	R^2^	Age p-value	Density p-value	Price p-value	Income p-value
2015	Rarefied Richness	<0.001*, 4 and 24, 6.689	0.527	<0.001*	0.099	0.444	0.831
	Evenness	0.011*, 4 and 24, 4.105	0.406	0.005*	0.623	0.569	0.457
	Shannon-Wiener Diversity Index	<0.001*, 4 and 24, 7.417	0.552	<0.001*	0.064	0.941	0.511
2016	Rarefied Richness	<0.001*, 4 and 31, 7.643	0.497	<0.001*	0.064	0.974	0.521
	Evenness	0.002*, 4 and 31, 5.547	0.417	0.002*	0.070	0.839	0.777
	Shannon-Wiener Diversity Index	<0.001*, 4 and 31, 7.4	0.489	<0.001*	0.048*	0.776	0.568

Each diversity measure was modeled with neighborhood age, housing density, home price, and median household income as predictor variables for both years. Overall model p-values, degrees of freedom, F-statistics, and R^2^ values are reported, as well as individual p-values for each of the predictors. All significant variables were negatively correlated with all diversity metrics. Variance inflation factors were less than 1.5 for all predictors across both years. Significant relationships are denoted in bold with an asterisk.

**Fig 5:**
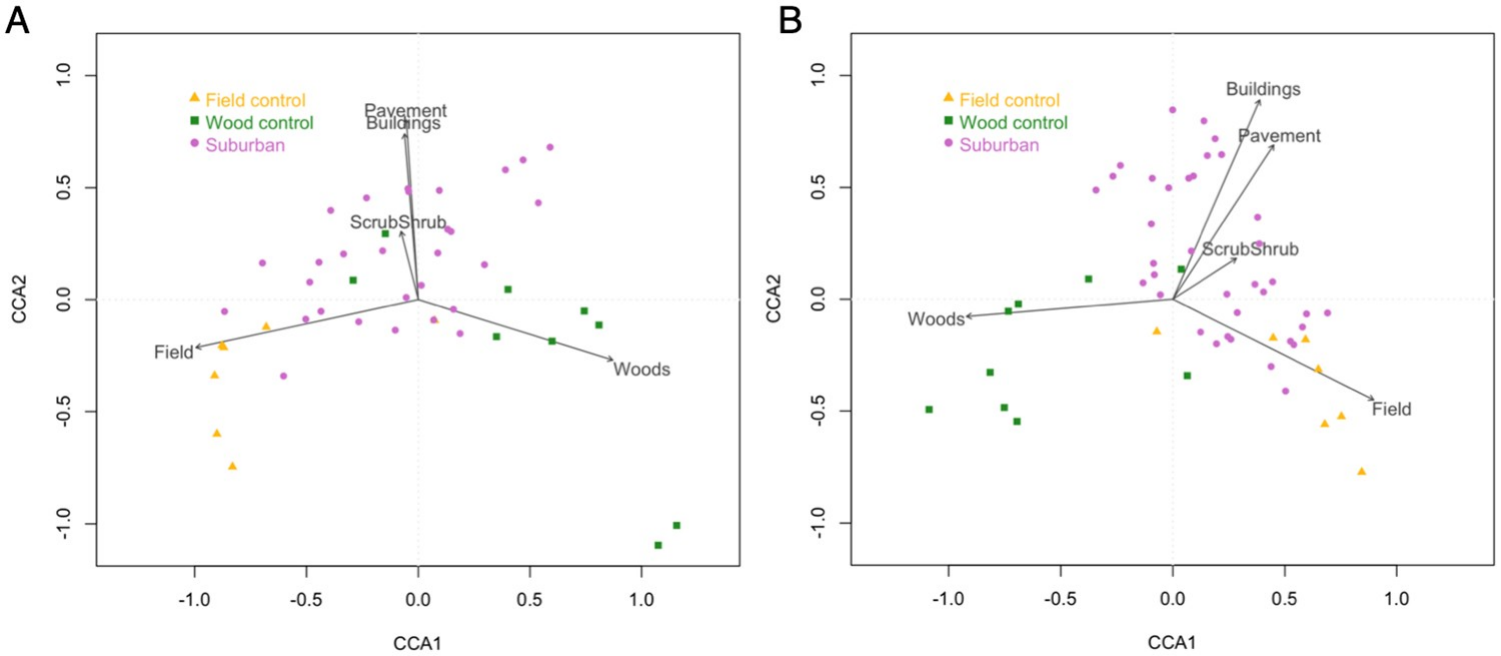
Partial Canonical Correspondence Analysis of mosquito assemblages and habitat classifications at each trapping site. For both PCCA figures, field control sites are represented by triangles, wood control sites are represented by squares, and suburban sites are represented by circles. Points are placed on the plot by the site’s mosquito community composition. Arrows for environmental factor of land-use classifications are overlaid, with the length of the arrow representing the strength of the relationship. (A) PCCA of the 2015 mosquito assemblage data. Environmental variables explained 35.9% of the total variation, with the first two axes explaining 86.4% of the environmental variation. (B) PCCA of the 2016 mosquito assemblage data. Environmental variables explained 26.6% of the total variation, with the first two axes explaining 88.4% of the environmental variation.

Given the differences in mosquito diversity and the relative abundance of *Ae*. *albopictus* among site types, the overall composition of mosquitoes also differed among site types. The environmental variables explained 35.9% of the total variation in the PCCA in 2015, with 86.4% of that variation explained by the first two CCA axes. Overall, field control sites and wooded control sites had distinct assemblages (Fig 5A), consistent with previous findings [21]. There did not appear to be any legacy effects of previous land use; that is, the suburban sites that were previously fields before development and those that were previously woods before development did not cluster together, but rather were fairly evenly dispersed throughout the range of suburban sites on the PCCA. Because of this lack of a legacy effect, suburban sites are shown in a single category on the PCCA for clarity. The suburban sites span across the field and wood habitat types on the CCA1 axis but separate out well on the CCA2 axis, suggesting that the pavement and buildings habitat components are important determinants of a suburban site’s mosquito assemblages in a manner unique to suburbia relative to grassland or forested sites (Fig 5A). Similar trends were seen in 2016, with the environmental variables explaining 26.6% of the total variation and 88.4% of that variation explained by the first two CCA axes. Again, there did not appear to be any legacy effects of previous land use among the suburban sites. The wooded and field control sites were distinct, and the suburban sites again separated out best on the CCA2 axis, suggesting that the buildings and pavement habitat components were most important in determining the suburban mosquito assemblage (Fig 5B).

To explicitly investigate neighborhood age in relation to the mosquito assemblage and land-use classification information in the PCCA, we plotted the CCA values for each site against neighborhood age. After adding a constant to all values so that all CCA values were non-negative, a power curve best fit the data for CCA1 values (residual SE = 0.72) and CCA2 values (residual SE = 0.61) in 2015, as well as for CCA1 values (residual SE = 0.59) and CCA2 values (residual SE = 0.80) in 2016 (S4 Fig). Based on these CCA regressions, the oldest suburban neighborhoods are in the upper right quadrant of the PCCA for 2015 (Fig 5A) and the upper left quadrant of the PCCA for 2016 (Fig 5B). In general, older neighborhoods tended to have mosquito community compositions more similar to those of woods than those of fields. Nonetheless, the suburban components of buildings and pavement are still the most important predictive land-use classification for the oldest neighborhoods, suggesting a unique suburban climax community. ANOSIM confirmed that the mosquito assemblage significantly differed between suburban sites, field control sites, and wooded control sites in 2015 (p = 0.001, R = 0.3061) and in 2016 (p = 0.003, R = 0.2449). *Aedes albopictus* was the most influential species, contributing 30% to 45.8% of the overall community dissimilarity in 2015 and 27.6% to 34.9% in 2016, depending on the site comparison (suburban vs. wood, suburban vs. field, or wood vs. field).

## Discussion

In comparing suburban neighborhoods with wooded and field sites, we found that the oldest neighborhoods have mosquito communities that are less species rich, less even, and less diverse when compared with either of the natural habitats. This decline in all diversity metrics as suburban neighborhoods age is concordant with a shift in species assemblage to a seemingly distinct suburban assemblage. The suburban assemblage is well correlated with the presence of impervious surfaces like pavement and buildings. Dominant among this suburban mosquito assemblage is the invasive species *Aedes albopictus*, whose abundance is positively correlated with neighborhood age.

Comparing diversity measures between control sites and suburban sites by their neighborhood age category did not yield significant differences between natural and suburban sites in 2015 (Fig 2). However, when incorporating older neighborhoods with the 2016 data, significant differences were found between the oldest neighborhood age category and both field and woodlot control sites in terms of rarefied richness, evenness, and Shannon-Wiener diversity (Fig 3). This shows that mosquito diversity in suburban neighborhoods becomes fundamentally different from that in undeveloped natural sites over time. Additionally, we performed linear regressions to assess the effect of neighborhood age, housing density, home price, and income on our three diversity measures. Variance inflation factors were less than 1.5 for all predictors across both years, indicating that there is not significant concern for collinearity among neighborhood age, housing density, home price, and median household income in the current study. Analysis of linear regressions revealed that neighborhood age was the only significant variable across both years for any diversity measure (Table 1), indicating that neighborhood age is the primary driver of mosquito diversity changes among suburban sites. However, a more appropriate way to analyze the changes that occur after suburban development is in looking beyond just diversity measures and instead to changes in species composition, which we accomplished with the PCCAs.

PCCA of the 2015 mosquito assemblage data shows that the mosquitoes collected at field control sites cluster together and are well defined by the field environment, with similar trends seen with the wooded control sites. In contrast, the mosquitoes collected at suburban control sites are best defined by pavement, buildings, and scrub/shrub environmental variables, suggesting that there is a distinct mosquito assemblage created as a result of common anthropogenic landscape components (Fig 5A). Similar results are seen in the PCCA of the 2016 mosquito assemblage data. The wooded and field sites are similar in their mosquito assemblages and separate out well based on their distinctive environments, while the mosquito assemblages at the suburban sites are best defined by the buildings, pavement, and scrub/shrub environmental variables on the opposite CCA axis (Fig 5B). When plotting CCA values against neighborhood age to explicitly investigate the effect of neighborhood age on mosquito communities, both CCA1 and CCA2 values for suburban sites in 2015 increased with neighborhood age toward a maximum value in the climax community (S4A and S4B Fig). Taken together, these plots show that the oldest neighborhoods are in the upper right quadrant of the PCCA (Fig 5A), correlating with the woods environmental variable on the CCA1 axis, and the pavement, buildings, and scrub/shrub variables on the CCA2 axis. For 2016, CCA1 values decreased with neighborhood age, while CCA2 values increased with neighborhood age (S4C and S4D Fig). This shows that the oldest neighborhoods in 2016 fall into the upper left quadrant of the PCCA (Fig 5B), again correlating with woods on the CCA1 axis, and suburban environmental factors on the CCA2 axis.

Overall, we found that as neighborhoods age, their mosquito assemblages shift somewhat from species associated with fields to those associated with woods. However, despite the potential shift from fine vegetation to coarse vegetation as a possible mechanism for this slight shift in mosquito assemblage as a neighborhood matures, pavement and buildings remain extremely important in determining suburban mosquito species assemblages with increasing neighborhood age. Indeed, the oldest neighborhoods did not recover their mosquito diversity and revert to a wooded species assemblage, but instead became increasingly uneven and less diverse, suggesting the suburban assemblage is different from that present in either natural habitat. A recent study has shown that urbanization caused not only functional diversity loss in bird communities, but also a loss of phylogenetic diversity, where evolutionary distinctiveness of species is lessened in urban areas [39]. It is hard to assess whether this holds for mosquito communities as mosquito phylogenies are currently greatly debated, but future work could investigate the potential for evolutionary impacts created by shifts to a suburban mosquito assemblage.

The most commonly encountered species in our study was *Ae*. *albopictus*, an invasive peridomestic species that thrives in artificial containers associated with anthropogenic land use [40,41], particularly in suburban habitats. The differences in mosquito assemblages seen when transitioning from natural habitats to suburban habitats are primarily driven by changes in the abundance of *Ae*. *albopictus*. While *Cx*. *erraticus* abundance was also significantly correlated with neighborhood age across both of the years in this study, *Ae*. *albopictus* more profoundly affected the suburban mosquito assemblage, as evidenced by the large difference between the two species in numbers of individuals caught as well as the individual contributions by *Ae*. *albopictus* of 27.6% to 45.8% to the overall community dissimilarity between site types detected in the SIMPER analysis. In general, the most conspicuous pattern seen throughout the study is that as neighborhoods age, their mosquito communities become less species rich, less even, less diverse, and more dominated by the introduced species *Ae*. *albopictus*.

A possible mechanism for the abundance of *Ae*. *albopictus* in suburban areas is the increased availability of container larval habitat when compared to rural counterparts. Mosquitoes as a whole utilize a wide variety of larval habitats, with each individual species best adapted to a specific type of larval habitat. These aquatic habitats can include both permanent and temporary bodies of water such as ephemeral rainwater pools in fields and woodlands, tree holes, floodwater pools, ditches, ponds and marshes, sluggish streams and stream overflow pools, and man-made containers [30]. With the exception of man-made containers, most of these larval habitat types are likely decreasing in availability as land-use shifts from undeveloped to highly suburbanized. A recent study [42] found significant increases in 3^rd^ to 4^th^ instar *Ae*. *albopictus* larvae in suburban areas, and, given significant differences in container availability between urban and rural areas, the study concluded that urbanization generally increases larval habitats for *Ae*. *albopictus*. This could, however, be complicated by socioeconomic factors, as *Ae*. *albopictus* prevalence varies by level of abandonment, with poor neighborhoods having more of this species than wealthier neighborhoods even within highly urbanized city centers [43]. While neither home price nor median household income as proxies for socioeconomic status significantly affected mosquito communities in the present study, larval habitat may be a key factor in the abundance of *Ae*. *albopictus* that was observed in suburban areas [42]. Further research is warranted to categorize the differences in container larval habitat in developed areas versus natural areas, as this may help guide land development policies or homeowner behavior to benefit vector management.

Aside from their ability to easily exploit artificial containers, previous work has also shown that *Ae*. *albopictus* is an edge species, flourishing at ecotones between field and wooded habitats in natural areas [21]. Given that suburban areas are fragmented, heterogeneous landscapes where small plots of grasslands (lawns) are interspersed with trees and other medium to coarse vegetation, it is possible that suburban neighborhoods are functioning as large spaces of contiguous edge habitats, allowing edge species such as *Ae*. *albopictus* to thrive. This trend for an abundance of edge species in suburban areas has previously been documented in ants [44]. In that study, the authors found an increase in ant species diversity in suburban areas, which they attributed to the presence of forest species that are rare in fully open areas, but likely to be present at ecotones, which the suburban landscape created. Despite similar attribution of suburbia as an edge habitat causing changes in species diversity, results from the ant study [44] do not agree with the current study’s findings of decreased overall diversity, likely because of the presence and dominance of the invasive *Ae*. *albopictus*. Previous studies in bird communities have found that as neighborhoods age, species diversity decreases and the community composition shifts from primarily native species to heightened dominance of invasive species [14]. This aligns with the prediction that in areas of anthropogenic disturbance, the intermediate disturbance hypothesis only holds when native species are considered, as invasive species thrive in disturbed areas [45]. Our findings also support this prediction, as demonstrated by the increasing dominance of *Ae*. *albopictus* as suburban neighborhoods age.

Our study was potentially limited by the choice to sample exclusively with a dry ice baited CDC light trap with the light removed. Each trapping method has its own biases, and one of the most prominent criticisms against the CDC light trap is its underrepresentation of *Ae*. *albopictus* [46]. Despite this bias against *Ae*. *albopictus*, it was our most abundant collected species. As traps were left overnight for approximately 16 hours, the 1kg of dry ice did not last throughout the entire trapping period. However, since traps were placed in the late afternoon, it is reasonable to assume that day-biting, crepuscular, and nocturnal mosquitoes were all potentially sampled for at least part of their host-seeking period before the dry ice sublimated. Removing the light had the benefit of decreasing bycatch, but it likely decreased the attractive range of the trap, particularly for light-attractive species. Since the attractive range of the CO_2_ baited CDC light trap is less than 15m even with the light intact [47], we anticipated our attractive power to be at a short range. Active attractive range of the CDC light trap is limited, but common mosquitoes in our study areas have been shown to disperse 300m to 1km [48], with *Ae*. *albopictus* having a daily mean distance traveled of 119m [49]. Housing density within 100m of a trap, and therefore within the average daily distance traveled by mosquitoes, ranged from 1 (in an area of active new construction) to 59 houses (in an extremely dense area with very small yards), with an average of about 20 houses per 100m across both years of trapping. This indicates that our adult trapping could have sampled from mosquitoes produced in larval habitats in many of the neighboring yards and surrounding areas, despite trapping at a single house per neighborhood. Although the limitations of trap choice and sampling scheme were not prohibitive in this study, future research could use varied mosquito sampling techniques and increased intra-neighborhood replication to further investigate questions of suburban succession of mosquito communities.

Previous studies have shown that mosquito assemblages in low diversity urban areas tend to be dominated by vector species of concern for human diseases [50]. Our results suggest that with steadily increasing land-use change and the particular fervor of suburban development, mosquito species assemblages will be altered and *Ae*. *albopictus*, a known vector, will become more common. These community level changes can significantly alter vector-borne disease risk for many pathosystems. *Aedes albopictus* readily bites humans, and is a competent vector of myriad pathogens of global human health concern, including dengue virus, West Nile virus, chikungunya virus, and Zika virus [41,51], as well as pathogens of focal concern in North Carolina, including La Crosse virus [52,53]. The overall community simplification and increasing abundance of *Ae*. *albopictus* in highly suburbanized areas make human-vector interactions more likely, heightening the potential for transmission of these concerning vector-borne diseases through both greater vector population size alone, and an increase in host biting rate due to proximity for contact [54]. In light of the overwhelming success of *Ae*. *albopictus* in established suburban areas, further research is warranted to elucidate how to mitigate the potentially devastating effects of suburban development favoring competent vector species.

## Supporting information

S1 Fig Trends in research on mosquito diversity changes during urbanization. Number of peer-reviewed publications by year on topics “urbanization” and “diversity,” “urbanization” and “mosquito,” as well as “urbanization” and “mosquito” and “diversity.” Citation reports accessed from Web of Science Core Collection on September 3, 2018.
(TIF)

S2 Fig Species accumulation curves based on random sampling. Species accumulation curves for (A) the 2015 data and (B) the 2016 data. Vertical bars represent 95% confidence intervals.
(TIF)

S3 Fig Log abundance of *Culex erraticus* decreases with log neighborhood age. Control field and woodlot sites were excluded from this analysis to focus on the effect of suburban development as these areas become further established. (A) In 2015, a significant negative correlation was noted (p = 0.001, df = 1 and 27, F = 12.54, R^2^ = 0.317, ρ = -0.544). (B) In 2016, a similar significant negative correlation was noted (p < 0.001, df = 1 and 34, F = 19.66, R^2^ = 0.366, ρ = -0.613).
(TIF)

S4 Fig Comparison of suburban site CCA values versus neighborhood age. CCA values for the suburban sites were plotted against neighborhood age to explicitly assess the effect of time on the suburban mosquito assemblage. (A) CCA1 values from 2015 were best modeled by a power curve (residual SE = 0.72). (B) CCA2 values from 2015 were also best modeled by a power curve (residual SE = 0.61). (C) CCA1 values from 2016 best fit a power curve with a negative relationship (residual SE = 0.59), while (D) CCA2 values from 2016 best fit a power curve with a positive relationship (residual SE = 0.8).
(TIF)

S1 Table Relationship between neighborhood age and mosquito species abundance. The five most prevalent species during both of the trapping years were assessed for their relationship with neighborhood age. Due to differential numbers of species caught in each trapping year, a total of six mosquito species were assessed. P-values, degrees of freedom, and F-statistics for the linear regressions of log abundance versus log neighborhood age are presented, with significant relationships denoted in bold with an asterisk. R^2^ and Spearman’s coefficient are given for significant relationships.
(TIF)
