# Supplementary figures and images for: Simplification of vector communities during suburban succession
Source: PLoS One. 2019 May 1;14(5):e0215485. doi: 10.1371/journal.pone.0215485 (PMC6493735; doi:10.1371/journal.pone.0215485)

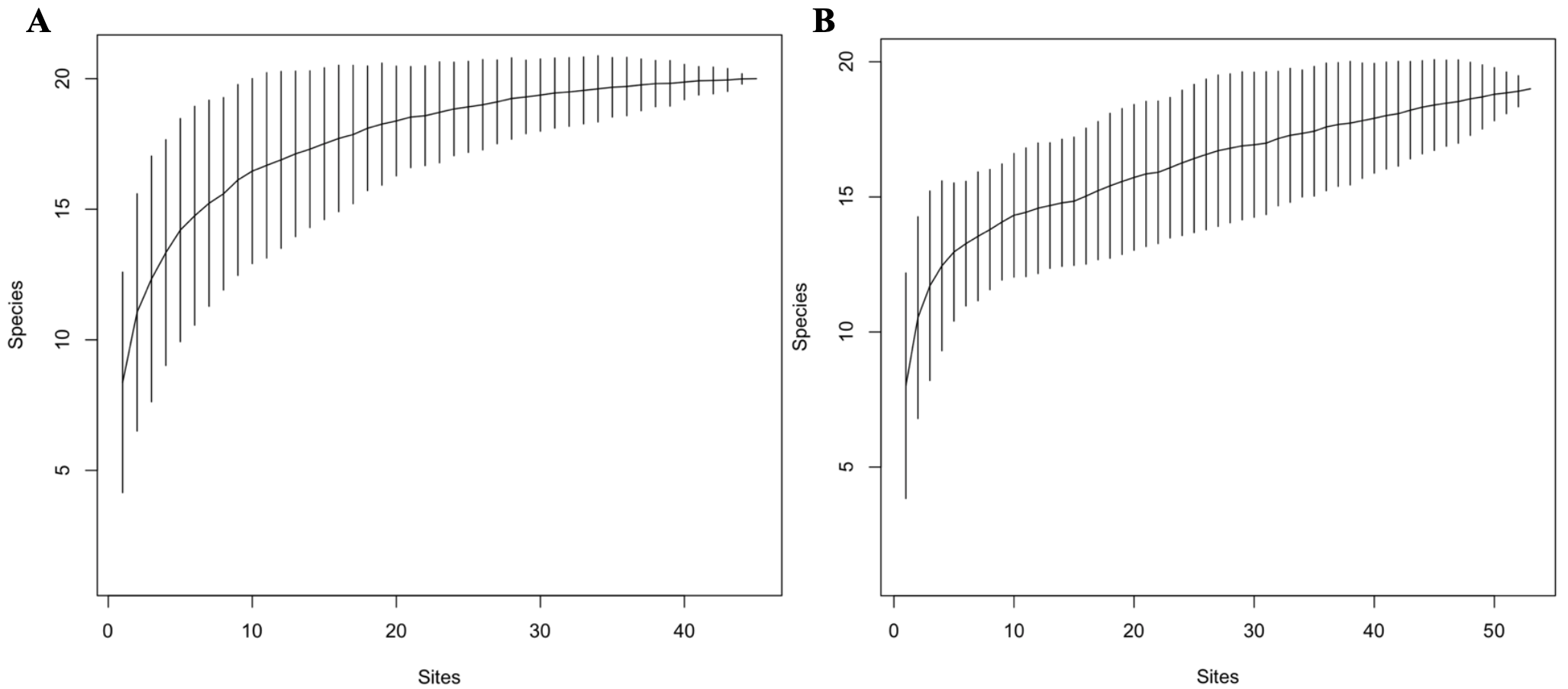

Supplement: S2 Fig — Species accumulation curves for (A) the 2015 data and (B) the 2016 data. Vertical bars represent 95% confidence intervals. (TIF) [file pone.0215485.s002.tif]

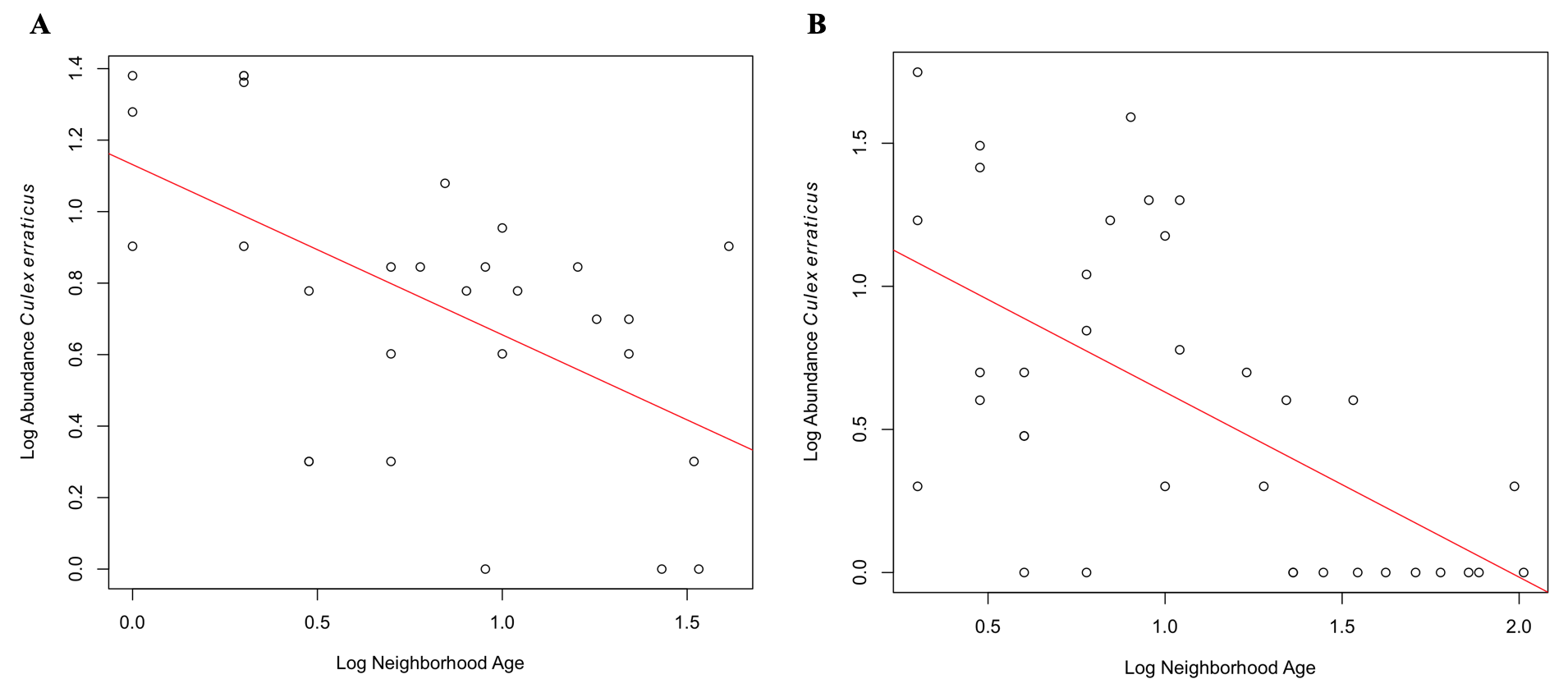

Supplement: S3 Fig — Control field and woodlot sites were excluded from this analysis to focus on the effect of suburban development as these areas become further established. (A) In 2015, a significant negative correlation was noted (p = 0.001, df = 1 and 27, F = 12.54, R2 = 0.317, ρ = -0.544). (B) In 2016, a similar significant negative correlation was noted (p < 0.001, df = 1 and 34, F = 19.66, R2 = 0.366, ρ = -0.613). (TIF) [file pone.0215485.s003.tif]

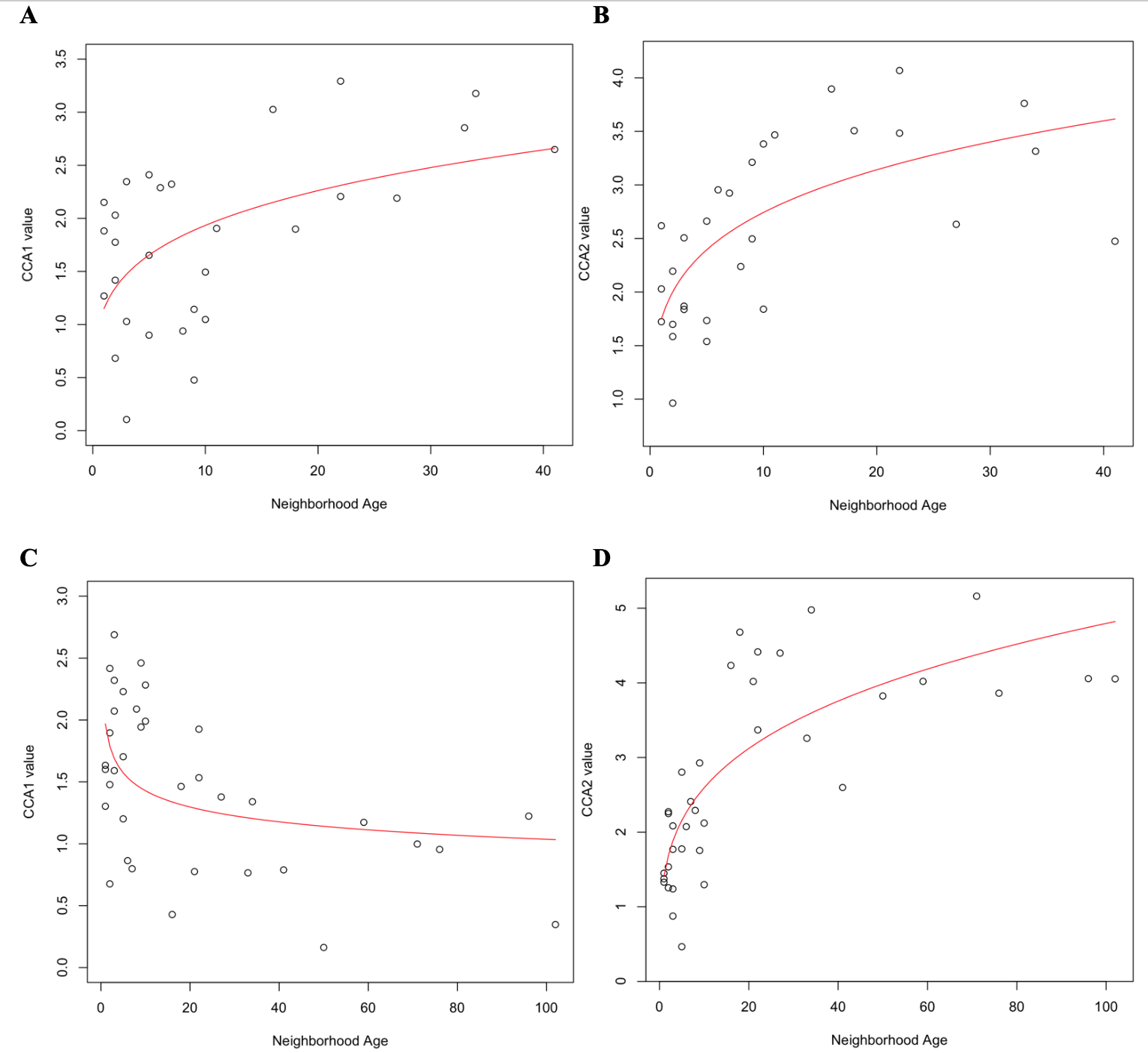

Supplement: S4 Fig — CCA values for the suburban sites were plotted against neighborhood age to explicitly assess the effect of time on the suburban mosquito assemblage. (A) CCA1 values from 2015 were best modeled by a power curve (residual SE = 0.72). (B) CCA2 values from 2015 were also best modeled by a power curve (residual SE = 0.61). (C) CCA1 values from 2016 best fit a power curve with a negative relationship (residual SE = 0.59), while (D) CCA2 values from 2016 best fit a power curve with a positive relationship (residual SE = 0.8). (TIF) [file pone.0215485.s004.tif]

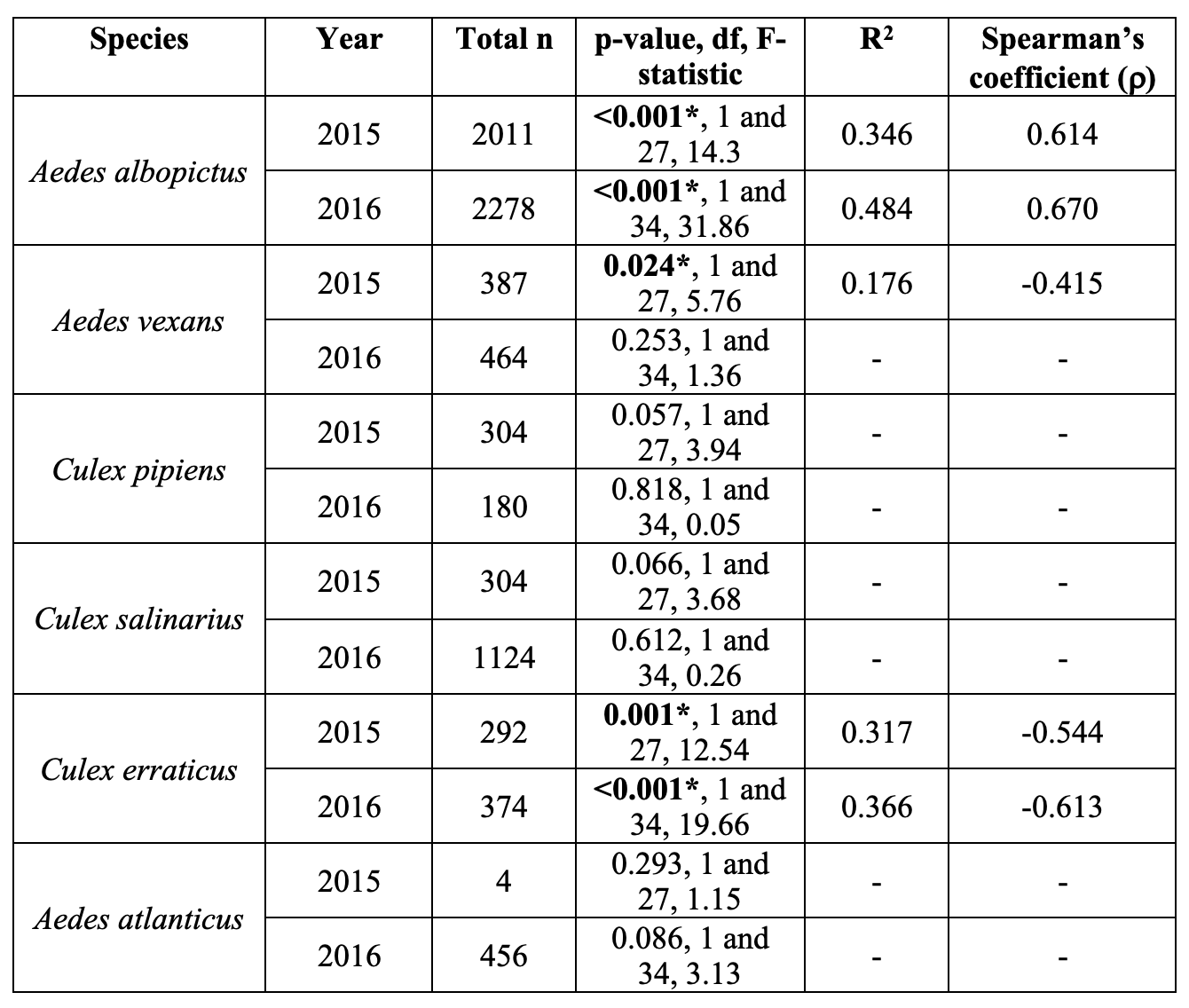

Supplement: S1 Table — The five most prevalent species during both of the trapping years were assessed for their relationship with neighborhood age. Due to differential numbers of species caught in each trapping year, a total of six mosquito species were assessed. P-values, degrees of freedom, and F-statistics for the linear regressions of log abundance versus log neighborhood age are presented, with significant relationships denoted in bold with an asterisk. R2 and Spearman’s coefficient are given for significant relationships. (TIF) [file pone.0215485.s005.tif]
